# Axons emanating from dendrites: phylogenetic repercussions with Cajalian hues

**DOI:** 10.3389/fnana.2014.00133

**Published:** 2014-11-18

**Authors:** Lazaros C. Triarhou

**Affiliations:** Laboratory of Theoretical and Applied Neuroscience, Department of Educational Policy, University of MacedoniaThessalonica, Greece

**Keywords:** neuron polarity, Pedro Ramón, Santiago Ramón y Cajal, evolution, ontophylogeny

## Introduction

The observation that some axons, in hippocampal pyramidal neurons, emanate from dendrites rather than the somatic envelope, and the contributory synaptic input privilege (Thome et al., 2014) was highlighted as a new dimension in understanding input-output transformations (Kaifosh and Losonczy, 2014). Applying state-of-art immunocytochemical, neurophysiological, and computational methods to Thy1-DsRed transgenic, wild-type C57BL/6J mice, and Wistar rats, Thome et al. (2014) found that one-half of CA1, one-third of CA3, and one-fifth of subicular pyramidal cells feature axon-carrying dendrites (ACDs).

Two-photon glutamate uncaging onto ACD spines led to excitatory postsynaptic potentials (EPSPs) and output action potentials (APs) at lower activation Δ-thresholds compared to nonACDs (Thome et al., 2014). EPSPs arising at nonACDs pass the soma and attenuate before reaching the axon initial segment (AIS), whereas in ACDs the electrotonic distance between excitatory synapses and the AP trigger zone is shorter. ACDs have a higher propensity to generate active dendritic d-spikes, primarily mediated by voltage-gated Na^+^ channels, and consistent with a lower functional density of A-type K^+^ channels (Kaifosh and Losonczy, 2014).

A question outstanding is to what extent this dendrito-axonal particularity is evolutionarily regulated across species (Kaifosh and Losonczy, 2014). I weave data from comparative studies to illuminate its phylogenetic basis.

## Mammals to insects

Axons occasionally emanate from dendrites in neocortical pyramidal cells (Peters et al., 1968; Sloper and Powell, 1979), midbrain dopamine neurons (Häusser et al., 1995), GABA interneurons targeting CA1 pyramidal dendrites (Martina et al., 2000), and neuroendocrine cells (Herde et al., 2013).

Generalities regarding mechanisms are greater at lower than higher levels; to decipher connections leading to motor patterns, neurons and networks are analyzed in molluscs, crustaceans, lampreys, worms, amphibians, and arthropods (Burrows, 1992). Intracellular recordings from insect motoneurons reinforce and enlarge on synaptic integration principles from the mammalian spinal cord (Simmons and Young, 2010).

In the flight motoneurons of the locust, which control the forewing or hindleg muscles, the dendritic arbor and the axon are combined into one process, attached to the soma by a slender stalk. The soma is not in the signal flow pathway. While axonal spikes are 100 mV in amplitude and <1 ms in duration, somatic signals conduct passively (10 mV lasting several ms). Dendrites with voltage-sensitive channels in their membrane can boost the amplitude of input signals. Thus, when an electrode is inserted into the motoneuron dendritic fan, near the axon origin rather than the soma, EPSPs rise and fall more sharply (Burrows, 1992; Gabbiani et al., 2002; Simmons and Young, 2010).

## Ramón and Cajal

Axons arising from dendrites in vertebrates and invertebrates were documented by Santiago Ramón y Cajal and his younger brother Pedro Ramón. In 1897 Cajal realized that “contrary to the general opinion, the soma does not always participate in the conduction of received nerve impulses; the afferent wave is sometimes propagated directly from dendrites to axon” (Ramón y Cajal, 1937). Accordingly, Cajal substituted his principle of *dynamic polarization* with *axopetal polarization*, whereby soma and dendrites conduct the waves of the nervous excitation toward the axon, which in turn carries impulses toward its terminal arborizations. “Currents flowing into the axon do not pass through the soma except when the latter is between the dendritic and the axonal apparatus” (Ramón y Cajal, 1897, 1937). Given the integrative processes of neuronal dynamics, one gathers that, when the axon fires ahead of the soma, it must emerge from a dendrite (Häusser et al., 1995; Yuste and Tank, 1996).

**Figure 1:**
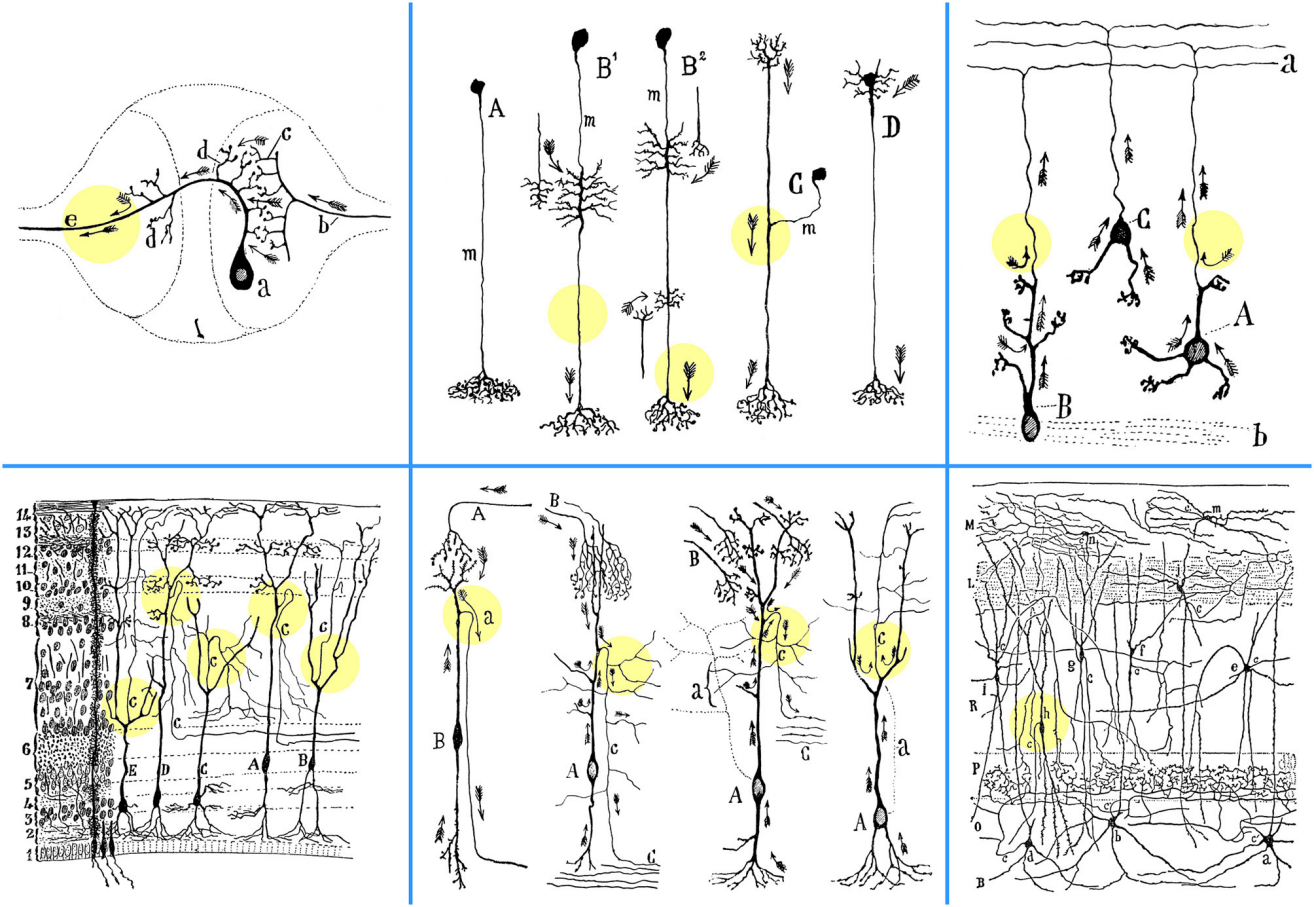
**Depiction by Cajal of the emergence of axons from dendrites in various anatomical settings. Upper left:** Sketch composed by Cajal, based on diagrams previously drawn by Mihály von Lenhossék and Gustaf Retzius, of the contacts between a sensory and a motor neuron in the ganglion of the earthworm *Lumbricus agricola* (Ramón y Cajal, 1899, p. 91; 1984, p. 143). Considered in this light, the “axopetal theory” is confirmed with all its details in the unipolar cells of invertebrates, also subsumed under the general functional dynamics of the vertebrate neurons. Abbreviations: *a*, crossed motoneuron; *b*, bifurcating sensory afferent fiber with collateral branches (*c*); *d*, the initial processes of the axon, acting as dendrites or receptive apparatus of motoneurons; *e*, motor axon. **Upper middle:** Scheme of diverse neuron types in the nervous system of insects. *A*, first type or amacrine cell; *B*^1^, *B*^2^, third type or cell equiped with two classes of appendices; *C*, second type or cell in T-branch; *D*, neuron with rudimentary somatic dendrites; *m*, neuronal shaft (*mango*), or indifferent intercalated segment (Ramón y Cajal et al., 1915). **Upper right:** Granule cells in the rat cerebellum (Ramón y Cajal, 1899, p. 102). *A*, granule cell in which the axon arises from a dendritic extension; *B*, another cell of the same type, the soma of which, however, lies in full in the white matter (*b*); *C*, granule cell whose axon rises from the soma; *a*, molecular layer. **Lower left:** Frontal section through the optic lobe of the chameleon, impregnated with the Golgi method (Ramón y Cajal, 1899, p. 88; 1984, p. 138). *A*, *C*, *D*, variants of *crosier* or *shepherd's crook* cells; *B*, *E*, cells with ascending axon (according to Pedro Ramón). Numbers on the left indicate the order of layers, from deep to superficial. **Lower middle:** Four examples of neurons (left to right), where an axon emanates from a dendrite. “In that instance, the axon issues from the distal end of a long dendrite, often after it has already ramified repeatedly, and from the point of view of impulse conduction, the soma is no more critical than the dendrites; it is merely the place that gives rise to dendritic trunks, harbors the nucleus and other inclusions.”**First:** Course of currents in crosier cells (*B*) of the optic lobe of fish, amphibians, and reptiles, where the axon (*a*) originates from a dendrite far from the soma. This is explainable by the “theory of axopetal polarization” (Ramón y Cajal, 1937, p. 392). *A*, afferent optic fibers. **Second:** Crosier or crook cell in the optic lobe of the sparrow, impregnated with the Golgi method (Ramón y Cajal, 1899, p. 89; 1984, p. 139). *A*, soma; *B*, fibers derived from the retina; *C*, central white matter; *c*, axon. Arrows indicate the direction of current flow. **Third:** Crosier cells of the optic lobe of reptiles, after Pedro Ramón (Ramón y Cajal, 1899, p. 100; 1984, p. 163). *A*, soma; *B*, optic fibers; *C*, deep white matter; *a*, trajectory of axon (*c*) economized as a result of its emergence from the end of the ascending dendritic trunk, rather than directly from the soma. **Fourth:** Highly elongated fusiform cell with a peripheral axon in the reptilian optic lobe, according to Pedro Ramón (Ramón y Cajal, 1899, p. 101; 1984, p. 165). Arrows mark the currents that flow in the axon (*c*), saving conduction time by arriving from the superior region of the cell; *a*, trajectory of the axon economized by arising from a superficial dendrite above the soma. **Lower right:** A section through the hippocampus of a one-month old rabbit, impregnated with the Golgi-Cox method (Ramón y Cajal, 1968, p. 18). Note the axon (*c*) emanating from the basal dendrite of a displaced pyramidal cell (*h*). *B*, white matter; *O*, stratum oriens; *P*, pyramidal layer; *a*, *b*, *d*, cells of horizontal axon; *e*, *f*, cells whose short axon goes to the stratum radiatum (*R*); *g*, *h*, dislocated pyramidal cells; *i*, cell whose axon sends branches to the interpyramidal plexus; *j*, cell of the lacunose layer (*L*); *m*, *n*, small cells of the molecular layer (*M*). Colored circles were added to highlight the exact points at which the axons emerge from dendrites.

Dendrite-derived axons occur in unipolar neurons of invertebrate abdominal ganglia (Ramón y Cajal, 1899, chapter V, “Physiological inductions of neuron morphology, and connections”). The segment that connects the dendritic stem to the initial part of the axon was called *accessory process* by Retzius (Figure 1). Dendrite-derived axons were documented by Ramón and by Cajal in the *corpúsculos del cayado* (“crosier” or “shepherd's crook” cells), and in elongated fusiform cells of the optic lobe of birds, reptiles, amphibians, and fish (Ramón, 1890, 1891, 1896; Ramón y Cajal, 1891; Triarhou and del Cerro, 2008).

Dendritic axons are seen in retinal Dogiel cells, cerebellar granule cells, cortical Martinotti cells, and spinal motoneurons. In pyramidal cells of the murine, cavine, and leporine hippocampus Ramón y Cajal (1968) noted that the axon sometimes arises from the soma and at other times from a thick protoplasmic process. Van der Loos (1976) found that one-tenth of cortical pyramidal cells in rabbits become improperly oriented (“inverted”) during migration, their axons emanating from either the soma or apical dendrites, an arrangement not observed in properly-oriented cells.

Sensory neurons in vertebrate spinal ganglia are unipolar, with the axon and the axopetal process (that serves as dendrite) originating from a common somatic stem. In the spinal ganglia of amphibians, reptiles, birds, and mammals, sensory neurons undergo a transient phase of bipolarity, and subsequently metamorphose into unipolar; thus, the soma ceases to lie between the two types of processes, and, in dynamic terms, “it is now removed from the direct pathway between skin and spinal cord.” In some fish, spinal ganglia neurons retain their bipolarity in maturity (Ramón y Cajal, 1984).

The surmise of a privileged synaptic input channel in ACDs (Thome et al., 2014) resonates Ramón y Cajal's (1899, 1984) laws of *economy of conduction time*, of *saving cellular material* and of *economy of space* to explain the emission of axons from dendrites or the unipolar form of spinal ganglion cells in worms, molluscs, crustacea, and insects.

The ontophylogenetic metamorphosis of sensory neurons from bipolar to unipolar and the concomitant transformation of a primordial winding path into rectilinear yield reduced distances, and shorter times for impulses to travel and information to be transferred; according to Cajal, “such a unipolar form was attained in stages, be it by natural selection or other evolutionary forces.” This idea is in harmony with the finding that the atypical cadherin Fat3 ensures in retinal amacrine cell precurors a transition from bipolar to unipolar, as they migrate through the neuroblastic layer (Deans et al., 2011).

The extrasomatic origin of axons from dendritic trunks reflects a conservation of cytoplasm, i.e., avoiding “senseless” waste of tissue matter: “Impulses gathered by distal dendritic branches have no reason whatsoever to pass through the soma; instead, they flow directly to the axon” (Ramón y Cajal, 1899).

## Cytoskeletal prompts

After axotomy of reticulospinal neurons in lampreys (*Petromyzon marinus*), sprouts with axon-like properties emanate from dendrites (Hall et al., 1989). Dendrite-derived axons (“dendraxons”) were reported after axotomy in *α*-motoneurons of cat spinal cord (level L7) (Lindå et al., 1985). Novel “dendrite-derived supernumerary axons” can be functional, as suggested by voltage-gated Na^+^ channel distribution and synaptophysin and glycoprotein SV2 expression, molecular prerequisites for AP propagation and neurotransmitter release (Meehan et al., 2011). Axotomized spinal commissural interneurons (level C3) also form *de novo* axon-like processes from distal or proximal dendrites or from the original axonal pole (Fenrich et al., 2007).

In dissociated and organotypic cultures of mouse hippocampus, proximally axotomized neurons yield new axons, with dynamic growth cones, from the tip of pre-existing basal or apical dendrites; in distal axotomies, axons regrow from their central stump. Axons growing from dendrites of severed CA1 pyramidal neurons *ex vivo* can establish functional synapses (Gomis-Rüth, 2007). In short, neurons retain a plasticity of polarity in development and maturity.

Axonal identity in developing and mature neurons is specified by microtubules; their stabilization with taxol, which decreases the catastrophe rate, induces multiple axon formation from differentiated dendrites (Gomis-Rüth et al., 2008). Microtubule stabilization triggers a positive feedback loop that includes changes in actin dynamics and axonal transport, complementary regulators of neuronal polarization. Signaling cascade molecules that control microtubule stability, such as glycogen synthase kinase-3 (GSK3) and collapsin response mediator protein-2 (CRMP2), play an active role in axonal determination and regrowth (Sweet and Firestein, 2008). Extracellular cues orchestrate the intracellular signaling that leads to axon-dendrite polarization in development (Barnes and Polleux, 2009). GSK3β is a critical regulator of polarity; GSK3β inhibition or depolymerization of the actin cytoskeleton converts immature dendrites into axons.

## Spongy origins

Neuronal networks are shaped by natural selection (Simmons and Young, 2010). Neurons are thought to have originated early in the evolution of eumetazoans, some 700,000,000 years ago, after diverging from sponges; the latter, a more ancient phyletic lineage, lack true neurons, a cnidarian-bilaterian synapomorphy (Richards et al., 2008). Nonetheless, the sponge *Amphimedon queenslandica* developmentally expresses, in migrating “globular” subepithelial cells destined to populate the larval epithelium, the proneural basic helix loop helix (bHLH) gene *AmqbHLH1*, coupled with Notch-Delta signaling, in a manner that resembles conserved molecular mechanisms of primary bilaterian neurogenesis. *AmqbHLH1* later duplicated to produce the *atonal*/*neurogenin*-related bHLH gene families, with strong proneural activity in *Xenopus* and *Drosophila* (Ma et al., 1996; Kim et al., 1997). Thus, eumetazoan neural differentiation and the bilaterian neurogenic circuit were probably added to an already established cell-determination program operational in the metazoan stem lineage, generating an ancient sensory cell type.

*Amphimedon*'s globular cells express components of what became the eumetazoan postsynapse, demonstrating that potential precursor components of neurons might be available in an ancestor which preceded true neurons. Globular cells have protruding apices receptive to environmental stimuli and a vesicle-packed cytosol (Richards et al., 2008). Therefore, early metazoans already possessed *proto-neural cells*, whose function plausibly entailed a primeval *output* (molecular secretion) in response to a sensory *input* (environmental stimuli).

## Synthesis

Ramón y Cajal (1995) postulated that the appearance of impulse polarity during animal evolution coincided with that of the nervous system itself, simply a consequence of tissue differentiation into sensory (skin and sense organs), and motor surfaces (glands and muscles).

Neurons common to all nervous systems must have evolved from an ancestral proto-cell, the properties of which remain a subject of speculation. Present-day neurons were necessarily predated by more rudimentary forms, intermediate in the genesis of their elaborate genetic, metabolic, and structural systems. A common ancestor of all neurons may not have differed much from some present-day sponge cells, its host organism having dwelled in an aquatic environment.

In today's neurons, information generally flows from dendrites to axons. In early evolution, however, a unipolar form (sensing) could have practically predated the bipolar form (sensory-motor). Arguments include: (1) sensory proto-neural cells had secretion as a primitive form of output, while motor output as an axonal function arrived later; (2) neuroendocrine and paracrine secretion systems are phylogenetically older than synaptic neurotransmitter systems; (3) dendrites may occasionally subserve input and output neuronal functions, by both receiving synaptic input and concomitantly releasing neurotransmitter (e.g., nigral dendrites). The advent of dendrites represents a watershed in neuron morphology and function.

Compared with crucial dendritic and somatic functions (commonalities include cytoplasmic organelles, enzymes, and biophysical properties), those fulfilled by the later elaborated axon and AIS are most intricate. This does not mean that dendrites could replace axons; but the compelling suggestion is that *dendrites preceded axons* in archaic neuron evolution and temporarily served, with the soma, a combined receptor-effector role.

One might suppose that, in phylogeny, ontogeny, and regeneration, a primitive process, strictly dendritic in form, could acquire, by progressive specification, the morphofunctional characters of the axis cylinder. In this hypothetical picture, unipolar proto-neural sponge cells gave rise to neurons. A unipolar form, with one process comprising dendrite and axon, was retained in later life forms, like insects. Bipolar forms evolved, as in fish. In mammals, an ontogenetic de-differentiation from bipolarity to unipolarity would confer a functional advantage, as highlighted by the important recent findings for hippocampal pyramidal neurons.

## Envoy

In Cajal's words, “obstacles in research can be overcome by a rational interpretation based on established facts of ontophylogeny; if we descend in the animal scale (worms, molluscs, crustaceans), or go back to early embryonic stages, we readily realize how the neuron assumed its form in vertebrates (amphibians, reptiles, mammals)” (Ramón y Cajal, 1899). A “triple-synthesis” was also one of the prime contributions of Christofredo Jakob: “Knowledge of the structure of the nervous system is reached by the following methods: comparative anatomy-embryology, development, and experimentally-induced degenerations” (Jakob, 1896); in other words, “looking at the brain, usually after a bit of tampering, and always with some kind of a microscope” (Van der Loos, 1976).

### Conflict of interest statement

The author declares that the research was conducted in the absence of any commercial or financial relationships that could be construed as a potential conflict of interest.
